# Surgery

**Published:** 1859-05

**Authors:** 


### SURGERY.

XVI.—*New Method of Treating Powder-wounds.* By PROF. BUSCH, of Bonn.

Instead of treating powder-wounds by the painful process of digging out each single grain of powder with the knife or needle, Professor Busch recommends the fomentation of the wounded part with a strong solution of corrosive sublimate (gr. v. to ℥j ;) this application produces an eczematous inflammation; some of the vesicles simply dry up, others form scabs. On removing such a scab, the grains of powder are seen to adhere to its under surface, and underneath it a newly-formed, spotless epidermis is found. The scabs and epidermis scales, together with the grains of powder, may then be scraped off with a spatula.

Any other strongly irritating application may be used with success; the solution of sublimate recommends itself the most, because in using it, the degree of irritation can be controlled pretty accurately, and because after the healing of the eczema produced by it, a white skin remains.—(*Virchow's Archiv. f. Pathol. Anatomie, Physiologie u. f. Klin. Med.*, xiv. 3, 4.)

XVII.—*On the Treatment of Inflammation by Digital Compression.* By PROF. VANZETTI, of Padua.

After having been several times successful in treating aneurisms by digital compression, Professor Vanzetti conceived the idea of applying this therapeutic method, which is so simple in execution, and at the same time so efficacious in its results, to the treatment of inflammation of the extremities.

The actual knowledge we possess on the pathological physiology of inflammation, permits the surgeon to preconceive what would be the effects of compression of the principal artery, the branches of which are distributed to the suffering part; the retardation of the circulation of the blood would soon be followed by a considerable diminution of the inflammatory symptoms. The results obtained by this method have been so satisfactory, that M. Vanzetti does not hesitate to adopt it as a usual means of treatment in preference to any other. Twelve to fifteen hours of intermittent compression are sufficient for obtaining a considerable amelioration; the patient can, in the majority of cases, make the pressure himself, after the surgeon has made it for one to two hours, and has shown him how to apply the fingers upon the course of the artery. If the hand, after having been applied for some time, commences to experience fatigue, the compression may be left off, and resumed again after the fingers have well rested.

Professor Vanzetti reports two interesting cases, which serve to support his observations: one of phlegmonous erysipelas of the arm, the other of arthritis of the wrist, in which the cure was promptly obtained by compression alone; and from these facts he draws the following conclusions :—

1. Digital compression cures inflammation quickly, by retarding the impulse of blood in the diseased part.

2. It can be applied by the patient himself, and should be continued until the pulse of the inflamed member becomes equal in frequency and intensity to that of the sound side.

3. In cases in which the inflammation is already too far advanced to be cured by resolution, arterial compression establishes suppuration more promptly; the mortified portions of skin and cellular tissue are separated quicker and more easily.

4. Digital compression is superior to other means employed at the present, with a view to retard and intercept the circulation in the thoracic extremities, on account of the finger being able to reach and compress the subclavian artery successfully, when the humeral or axillary artery are rendered inaccessible by the inflammation of the parts surrounding them, and the subclavian cannot be compressed by any mechanical apparatus.—(*Cenni sulla cura dell' inflammazione colla compressione digitale.* Venise, 1858; and *Archives Générales,* February, 1859.)

XVIII.—*On the Treatment of Burns by the Permanent Warm Bath.* By DR. PASSAVANT.

Dr. Passavant had an opportunity of testing his method of treatment in thirteen cases of burns of all degrees which were occasioned by an explosion in a fireworks factory, and were brought into the Hospital of Frankfort. All these cases were treated by permanent warm baths, or, if this was inapplicable, by fomentations of warm water. The water was renewed twice daily or oftener, if the suppuration was very copious, and was always kept at a temperature of 27° Réaumur. The apparatus employed were similar to those used by Professor Langenbeck in the treatment of amputations; if, after some weeks, the patients became fatigued by them, warm fomentations were used in their stead.

The first effect produced by the bath, was a considerable relief, and the pain, which was excessive at the beginning, soon ceased completely. The parched and hardened tissues, which were penetrated by the water, became soft, and the sloughing parts detached themselves with more facility. Besides, this mode of treatment protected the injured surface from all causes of irritation, and diminished the chances of purulent resorption. Cicatrization finally took place more rapidly, owing to the maintenance of a uniform temperature, and to the light compression exercised by the water upon the diseased tissues.—(*Deutsche Klinik,* 1858, 36, 38, and 39; and *Archives Générales,* February, 1859.)

XIX.—*Clinical Remarks on Dermoid Cysts.* By PROFESSOR LEBERT.

This treatise, which includes several new observations, is destined to complete a memoir presented by the author to the *Société de Biologie* in the year 1852, in which are collected mostly observations relative to dermoid tumors. These tumors may be developed either in a part of new formation, or in a normal pre-existing cavity; they are composed of elements identical with those of the tegumentary system, which are developed on the internal surface of a sac closed on all sides. We meet there with epidermis, papillæ, the proper tissues of the skin, sudoriparous and sebaceous glands, hairs with their bulbs and ordinary coverings, and cellulo-adipose tissues; the cyst contains fatty masses emanating from the sebaceous glands, detached hairs, and isolated epidermic lamellæ. In addition to this, its walls often include teeth, bones, and cartilages, which are mixed in most variable proportions with the dermoid products. These productions belong, according to Professor Lebert, to the class of plastic heterotopies, and not to inclusion.

These cysts are often seated in the subcutaneous cellular tissue; if this variety is observed in animals, it is found that these productions of hair, etc., are always analogous to those which are met with on their integuments in the normal state, and that the tumors augment each year at the time when their hair falls, and is replaced by new epidermoid appendages.

The subcutaneous dermoid cysts in man exist usually from the time of birth; their seat of predilection is on the surface of the superior eyelid or of the root of the nose; in the former place they are generally oval, while those of the root of the nose are oftener spherical.

These tumors grow but very slowly, and it is rare that they ever exceed the volume of a hazlenut. The skin which covers them glides easily upon them, but they adhere often to the deep tissues, a circumstance which serves to distinguish them easily from atheromatous cysts which adhere to the skin, and are displaced with it; they are a little more frequent in men than in women.

On extirpation they are found to adhere intimately to the periosteum; it is important not to leave this deep part in place, as it is not exfoliated in consequence of the suppuration, like most other cysts, but may give rise to an obstinate fistula, if left behind. It happened in two cases reported by M. Lawrence, that in laying open the fistulous passage he found on the bottom of it the remainder of a cyst, from which several hairs were growing; on the removal of this residue, a cure was quickly obtained.

Dermoid cysts of the external genital organs in women are not very rare; they are found especially in the ovaries, but sometimes also in the tissue of the uterus. If they are not very voluminous and rest stationary, they often give rise to no symptoms; and the same is frequently the case in the early stages of those which some time later manifest a tendency to greater development. If the physician is then consulted, they have commonly acquired already the size of a fist. They are met with especially on the right side, where their presence causes a pain or a vague feeling of uneasiness in the abdomen; their subsequent development is generally accompanied by more pain than is the case in ordinary ovarian cysts, and is often attended with a copious leucorrhœa. Disorder of menstruation is not a constant symptom.

In most cases these tumors are for a long time of a rather hard or flabby consistence, and it is only at an advanced period that fluctuation is perceptible; the dermoid cyst has then become the seat of a serous exhalation; sometimes they contain only a serous liquid mixed with hairs and fat, and then an explorative puncture will be sufficient to establish the diagnosis.

Other symptoms, which in nowise differ from those of ordinary ovarian cysts, are referable to the compression of the organs contained in the true pelvis.

The cysts may empty themselves into the bladder, the rectum, or may perforate the abdominal walls, and thus evacuate their contents externally; the diagnosis is then easy. It must, however, be remarked, that they develop themselves not only in the ovary, but also in the retro-vesical cellular tissue; cases of this kind have been observed in men, and this possibility must always be kept in mind when we meet with a case in which urine containing hairs is voided. In more unfortunate circumstances, the cyst may empty itself into the peritoneum; Professor Lebert reports a case of this kind.

M. Jaczenky has seen a cyst of this description make its way into the vagina; an abscess opened at the bottom of the posterior cul-de-sac, and a hard body was discovered in this situation, which had troubled the husband of the patient very much during coitus, and had also excoriated the cheek of her last child during the act of labor; it was found to be a bone, furnished with two molar and two incisor teeth. The teeth were pulled out, but it was impossible to remove the bone which supported them.

In some cases, dermoid cysts, situated between the uterus and rectum may form an obstacle to the delivery of the child. In two cases of this kind, the expulsion of the child was followed by that of the cyst.

These cysts, even if they do not remain in the latent state, do not necessarily prove fatal, although this termination is much to be feared in consequence of the different perforations indicated above. They develop themselves principally at the period of puberty; in this respect they differ essentially from the subcutaneous cysts.

The treatment of dermoid cysts seated in the internal genital organs can often be but palliative; only if they show a tendency to open externally, interference with the bistoury or with the aid of caustics may be of use. If they have opened to the outside of the abdominal walls, or into the bladder, etc., the indications will be discerned without difficulty.

The memoir of Professor Lebert closes with the report of a case in which a dermoid cyst was situated in the anterior mediastinum.—(*Prager Vierteljahrschrift*, 1858, iv.; and *Archives Générales*, February, 1859.

